# Daily Remote Ischemic Conditioning Can Improve Cerebral Perfusion and Slow Arterial Progression of Adult Moyamoya Disease—A Randomized Controlled Study

**DOI:** 10.3389/fneur.2021.811854

**Published:** 2022-02-03

**Authors:** Jiali Xu, Qian Zhang, Gary B. Rajah, Wenbo Zhao, Fang Wu, Yuchuan Ding, Bowei Zhang, Wenting Guo, Qi Yang, Xiurong Xing, Sijie Li, Xunming Ji

**Affiliations:** ^1^Department of Neurology, Xuanwu Hospital, Capital Medical University, Beijing, China; ^2^Laboratory of Brain Disorders, Beijing Institute of Brain Disorders, Capital Medical University, Beijing, China; ^3^Department of Neurosurgery, Wayne State University, Detroit, MI, United States; ^4^Department of Neurosurgery, Munson Medical Center, Traverse City, MI, United States; ^5^Department of Radiology, Xuanwu Hospital, Capital Medical University, Beijing, China; ^6^Beijing Key Laboratory of Hypoxic Conditioning Translational Medicine, Xuanwu Hospital, Capital Medical University, Beijing, China; ^7^Department of Emergency, Xuanwu Hospital, Capital Medical University, Beijing, China

**Keywords:** moyamoya disease, stroke, remote ischemic conditioning, cerebral blood flow, arterial spin labeling (ASL)

## Abstract

**Background and Purpose:**

Moyamoya disease (MMD) is a complicated cerebrovascular disease with recurrent ischemic or hemorrhagic events. This study aimed to prove the safety and efficacy of remote ischemic conditioning (RIC) on MMD.

**Methods:**

In total, 34 patients with MMD participated in this pilot, prospective randomized controlled study for 1 year. 18 patients were allocated into the RIC group, and 16 patients accepted routine medical treatment only. RIC-related adverse events were recorded. The primary outcome was the improvement ratio of mean cerebral blood flow (mCBF) in middle cerebral artery territory measured by multidelay pseudocontinuous arterial spin labeling, and the secondary outcomes were the cumulative incidence of major adverse cerebrovascular events (MACEs), the prevalence of stenotic-occlusive progression, and periventricular anastomosis at 1-year follow-up.

**Results:**

In total, 30 of the 34 patients with MMD completed the final follow-up (17 in the RIC group and 13 in the control group). No adverse events of RIC were observed. The mCBF improvement ratio of the RIC group was distinctively higher compared with the control group (mCBF_−whole-*brain*_: 0.16 ± 0.15 vs. −0.03 ± 0.13, *p* = 0.001). Stenotic-occlusive progression occurred in 11.8% hemispheres in the RIC group and 38.5% in the control group (*p* = 0.021). The incidence of MACE was 5.9% in the RIC group and 30.8% in the control group (hazard ratio with RIC, 0.174; 95% CI, 0.019–1.557; *p* = 0.118). No statistical difference was documented in the periventricular anastomosis between the two groups after treatment.

**Conclusions:**

Remote ischemic conditioning has the potential to be a safe and effective adjunctive therapy for patients with MMD largely due to improving cerebral blood flow and slowing the arterial progression of the stenotic-occlusive lesions. These findings warrant future studies in larger trials.

## Introduction

Moyamoya disease (MMD) is a complicated cerebrovascular disease, mainly involving children or young adults characterized by stenosis or occlusion at the bifurcation of the circle of Willis and proliferation of basal collaterals (1). Chronic cerebral hypoperfusion can make patients with MMD suffer from ischemic stroke, transient ischemic attack (TIA), cognitive impairment, and even intracranial hemorrhage (2).

The mechanism of MMD has not been fully elucidated, thereby there is no specific medication for MMD. Antiplatelet therapy is utilized to prevent ischemic stroke and TIA, however, it is controversial for patients with MMD, because antiplatelet agents may increase the risk of intracranial hemorrhage in patients with MMD (3). Revascularization surgery has been shown to improve cerebral perfusion and reduce cerebrovascular events for patients with MMD (4–6), nevertheless, complications like hyperperfusion syndrome, postoperative stroke can occur and may lead to neurological deterioration (7).

Remote ischemic conditioning (RIC) is a non-invasive approach protecting the brain by repeated ischemia-reperfusion on the upper limbs. Neuroprotective factors were produced by the stimulus of RIC and these factors conferred a protective effect on remote target organs (8). It has been confirmed that RIC can improve cerebral perfusion by promoting angiogenesis and arteriogenesis in ischemic animal brains (9–11). In addition, daily RIC is a promising technique to ameliorate injury caused by chronic cerebrovascular diseases such as intracranial atherosclerotic stenosis (ICAS), small-vessel disease (12, 13). Meng et al. revealed that RIC could reduce recurrent stroke in patients with symptomatic ICAS by promoting cerebral perfusion (12). Wang et al. reported that daily RIC for 1 year could improve cognitive function and reduce the volume of white matter hyperintensities of small vessel disease (13). Recently, a single-arm study has reported that RIC relieved ischemic events and improved perfusion in ischemic patients with MMD (14). However, the efficacy of RIC for patients with MMD is still unknown in a randomized controlled setting. As far as we know, two randomized clinical trials about the efficacy of RIC for pediatric patients with MMD are in progress (NCT03546309 and NCT03821181) (15) and thus, we conducted a pilot randomized controlled study to explore the safety and efficacy of RIC treating adult patients with MMD to guide further trials.

## Methods

### Study Design

This was a single-center, open-label, prospective, parallel randomized study from 2019 July to 2021 February in patients with MMD with 1-year treatment at Xuanwu Hospital, Capital Medical University. This study was registered at clinicaltrials.gov with the unique identifier NCT04012268. This protocol was approved by the Ethics Committee of Xuanwu Hospital, Capital Medical University. All the subjects have provided written informed consent.

### Inclusion Criteria

Patients who participated in this study met all of the inclusion criteria: (1) Patients aged from 18 to 60 years; (2) All of the patients underwent digital subtraction angiography (DSA) and met the current diagnostic criteria recommended by the Research Committee on MMD of the Ministry of Health and Welfare of Japan in 2012; (3) Modified Rankin Scale (mRs) score ≤ 3; and (4) Informed consent obtained from the patient or acceptable surrogate patient.

### Exclusion Criteria

Subjects who met any of the exclusion criteria were excluded from this study: (1) patients with acute ischemic or hemorrhagic stroke within 3 months; (2) severe hepatic or renal dysfunction; (3) severe hemostatic disorder or severe coagulation dysfunction; (4) severe cardiac diseases; (5) patients with severe existing neurological or psychiatric disease; (6) patients with moyamoya syndrome caused by autoimmune disease, Down syndrome, neurofibromatosis, leptospiral infection, or previous skull-base radiation therapy; and (7) patients with completed or planned revascularization surgery.

### Randomization and Masking

The subjects diagnosed as MMD by DSA who had not been accepted for revascularization surgery were recruited. After baseline assessment, eligible patients signed informed consent. They were randomized in 1:1 ratio to accept either RIC plus routine medical treatment or routine medical treatment only with a computer-generated randomization code. The code was put into an opaque envelope. The investigators would number the eligible patients and open the envelope to determine the treatment plan. Investigators who assessed outcomes were blinded to the allocation.

### Sample Size

This was a pilot randomized controlled study. There were no parameters referred to estimate the sample size, and Hertzog (16) has shown that 10–20 subjects per group are adequate to evaluate the feasibility in a pilot study. In addition, Dobkin (17) has suggested that 15 subjects in each group are enough to evaluate whether a larger multicenter trial should be implemented. Thus, we aimed to recruit 15 patients per group, and finally, we recruited a total of 34 patients in this study.

### Procedures

Patients in the RIC group accepted bilateral upper limbs RIC performed by an autocontrol device (patent no.: ZL200820123637.X, China), including five cycles of inflating and deflating for 5 min alternately twice daily for 1 year. The inflating pressure was 200 mm Hg. The investigators performed telephone follow-ups every month to assure patients insisting on this treatment. Considering the effect of single-time RIC could sustain 4 days (18), the compliance of 1 month would be considered substandard if the patient discontinued RIC treatment for consecutive 4 days of the month. Both groups accepted routine medical treatment that includes antiplatelet therapy, lipid-lowering therapy, controlling of vascular risk factors, and adjunct drug butylphthalide. Antiplatelet therapy was not available for asymptomatic and hemorrhagic patients in this study.

### Outcome Assessment

The primary outcome was the mean cerebral blood flow (mCBF) improvement ratio after 1-year treatment. Secondary outcomes were the progression of stenotic-occlusive lesion, the change of periventricular anastomosis, and the incidence of major adverse cerebrovascular events (MACEs). The outcomes of safety were the incidence of adverse events related to RIC and the change of hematologic indexes. Fasting blood of patients was collected and hematologic indexes were examined including white blood cell count (WBC), red blood cell count (RBC), hemoglobin (HGB), and platelet count (PLT). Alanine transaminase (ALT) and aspartate aminotransferase (AST) were examined to reflect hepatic function, creatinine (CREA), and urea nitrogen (UREA) were obtained to assess renal function. Creatine kinase (CK), a biomarker for reflecting skeletal muscle, was also tracked to monitor for injury. Multidelay pseudocontinuous arterial spin-labeling MR (PCASL-MR), time-of-flight MR angiography (TOF-MRA), and hematologic indexes collection were performed within 1 week before and 1 year after the treatment.

#### Baseline and Follow-Up Evaluations

Demographic characteristics, clinical history including ischemic stroke-related risk factors, symptoms, concomitant diseases, and routine medical treatments were recorded. All participants underwent 3T black-blood T1-weighted intracranial vessel wall imaging to exclude moyamoya syndrome (such as ICAS, vasculitis, and so on).

#### Cerebral Blood Flow Evaluation

Neuroradiologic outcomes were assessed by two experienced radiologists who were blinded to the allocation, the result would be obtained when a consensus was achieved. The images of cerebral blood flow (CBF) were acquired from PCASL-MR which were performed at 12 ± 0.5 months from baseline. A 3T imaging system (Verio, Siemens Healthcare, Erlangen, Germany) with a 32-channel head coil was applied. The PCASL images were obtained by an acquisition protocol with an acquisition time of 5 min and 40 s, post-labeling delay of 1,500/2,000/2,500/3,000 ms, echo time of 22.67 ms, 26 scan slices, slice thickness of 5 mm, and a 64 × 64 matrix resolution. Data of PCASL in DICOM form were converted to perfusion maps. The CBF map was processed by OsiriX (University of California, Los Angeles, USA). In each hemisphere, the middle cerebral artery (MCA) territory was divided into 10 regions according to Albert Stroke Program Early CT score (ASPECTS). Regions of interest (ROI) were drawn manually in each region of the MCA to determine the absolute CBF values in slices of the corona radiata and the basal ganglia (detailed ROIs were depicted in Supplementary Figure I).

Mean CBF was calculated by formula: mCBF_−hemisphere_ = CBF (M1 + M2 + M3 + M4 + M5 + M6 + insula + internal capsule + lentiform nucleus + caudate)/10; mean CBF of the whole brain (mCBF_−whole-*brain*_) was defined as the average value of mCBF in the left and right hemisphere of the patient. mCBF_−cortex_ was the average value of CBF values in bilateral M1-M6 and insula. The mCBF_−basal−ganglia_ was defined as the mean value of CBF values in bilateral internal capsule, lentiform and caudate. The improvement ratio of corresponding mCBF was calculated by the formula: the mCBF improvement ratio = (mCBF at 1 year–mCBF at baseline)/mCBF at baseline.

#### Evaluation of Periventricular Anastomosis and Arterial Lesion Progression

The periventricular anastomosis and arterial lesion progression were evaluated by 3T 3D TOF-MRA. All the subjects were examined on a 3-Tesla system (Magnetom Verio; Siemens Healthineers, Erlangen, Germany), a 32-channel head coil was used for signal reception at 3T. The parameters of 3D TOF-MRA included: axial plane, flip angle 20°, TR/TE = 22/3.99 ms, FOV = 180 × 180 mm^2^, slice thickness = 0.8 mm, and matrix = 320 × 320).

The periventricular anastomosis is a connection beginning at the perforating arteries ending at the medullary artery at the lateral corner of the frontal horn or body of the lateral ventricle. The periventricular anastomosis was classified into three types: lenticulostriate anastomosis, thalamic anastomosis, and choroidal anastomosis (19). Each type of anastomosis was recorded as “presence” for “positive” and “absence” for “negative” from TOF-MRA images at sagittal orientation.

Arterial progression could be confirmed according to following conditions: (1) compared to baseline, the stenotic or occlusive lesion extended to another segment of Willis circle (for example, lesion extended from MCA M1 segment to M2 segment); (2) lesion extended from proximal portion to distal portion of one segment (such as extending from proximal portion to distal portion of MCA M1 segment); (3) stenotic lesion developed as the occlusive lesion. Houkin's MRA score (20) was used to evaluate the status of the arterial lesion in the Willis circle at baseline and 1 year follow-up.

#### Major Adverse Cerebrovascular Events Evaluation

Major adverse cerebrovascular events include hospital admissions with the TIA, ischemic stroke, and hemorrhagic stroke in this study. Patients were required to record the adverse events and were telephoned by two experienced neurologists every 3 months.

#### Statistical Analysis

To compare the characteristics at baseline or follow-up of the RIC group with the control group, continuous variables described as mean ± SD or medians (IQRs) were analyzed by the independent Student's *t*-test or the Mann–Whitney *U* test; categorical variables described as proportions were analyzed by the χ^2^ test or the Fisher exact test using SPSS Statistics Version 23 (IBM Incorporation, Armonk, New York, USA). To further evaluate the effect of RIC on the progression of stenotic-occlusive lesion, the binary logistic regression analysis was used to determine the odds ratio (OR) with RIC. For comparing the cumulative incidence of MACEs at 12 months, the Cox proportional-hazards models were used. Interrater agreements of CBF measurements were evaluated by intraclass correlation coefficients (ICC). Consistency between two radiologists is considered good when the ICC value ≥ 0.75. *p* < 0.05 was considered to be statistically significant for all the tests. All the tests were 2-sided.

## Results

**Figure 1:**
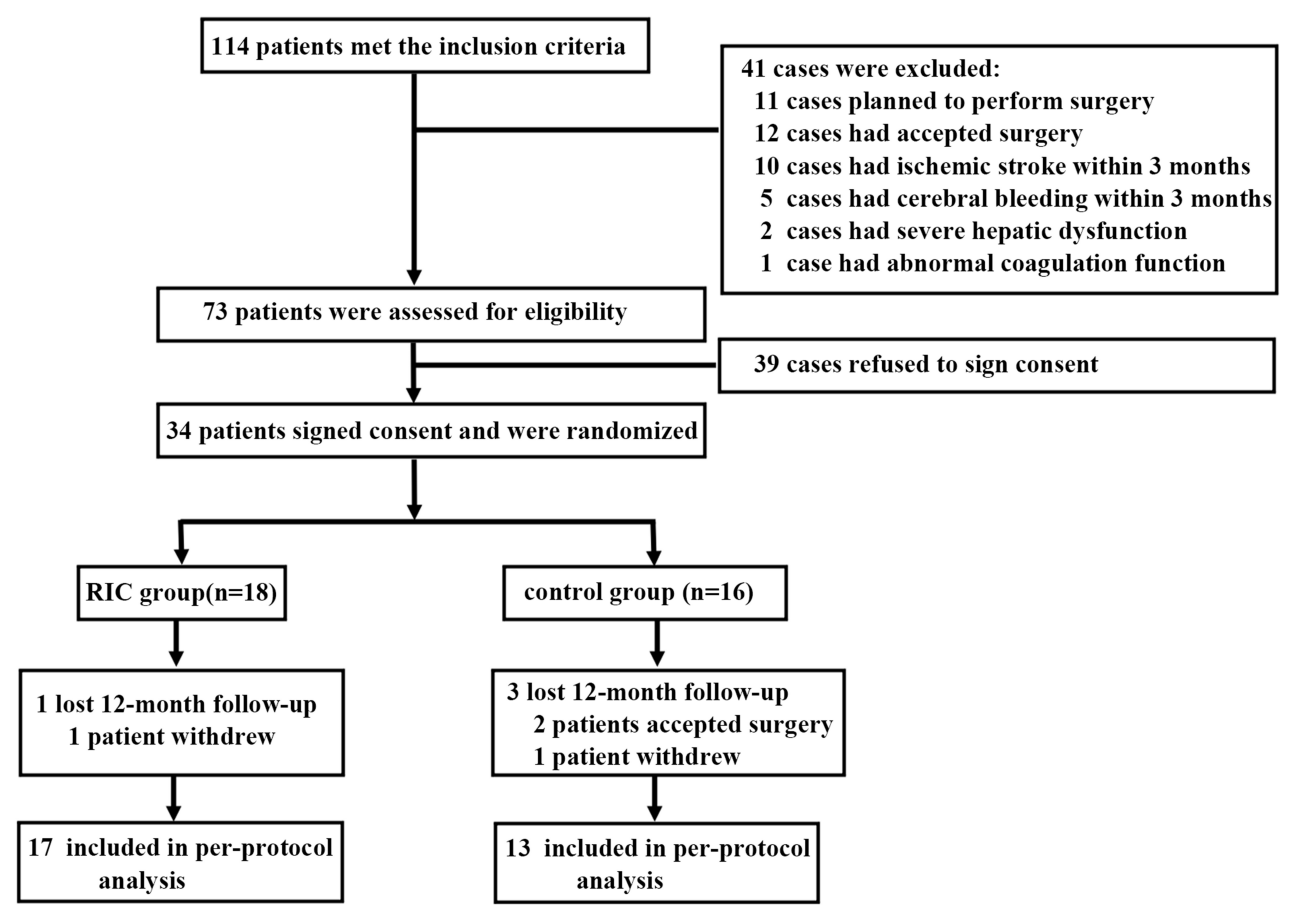
Flowchart.

After screening 114 patients diagnosed as adult MMD, 34 patients were recruited. In total, 18 patients were allocated to the RIC group and 16 patients were in the control group. A total of 17 patients in the RIC group completed the 1-year follow-up, while 13 patients in the control group completed the follow-up (Figure 1). Thereby, a total of 30 patients (60 hemispheres) were included in this per-protocol analysis. In the RIC group, all the patients complied ≥80% of the months.

### Baseline Characteristics

There were no significant differences between the two groups in demographic characteristics, symptoms, concomitant disease, medical treatment (Table 1), and hematologic indexes (Table 2). mCBF of the whole brain (RIC vs. control: 43.03 ± 8.70 vs. 47.06 ± 8.44 ml/100 g per min, *p* = 0.213), cortex (RIC vs. control: 45.07 ± 9.40 vs. 49.11 ± 10.23 ml/100 g per min, *p* = 0.278), basal ganglia (RIC vs. control: 38.28 ± 8.36 vs. 42.28 ± 10.30 ml/100 g per min, *p* = 0.243) were all not significantly different between the two groups at baseline (Table 2). In addition, no significant difference was shown in any type of periventricular anastomosis between the two groups (detailed characteristics were listed in Table 3).

**Table 1:** Baseline characteristics of the patients with moyamoya disease (MMD).

**Characteristic**	**Control** (N1^*^= 13)	**RIC** (N1^*^= 17)	***P*** **-value**
Male sex—no./total no. (%)	4/13 (30.8)	5/17 (29.4)	1.000
Age—yr	36.0 ± 10.7	39.1 ± 10.1	0.422
**Suzuki stage**			
Median	3	3	0.891
Range	2–4	2–4	
**Symptoms—no./total no. (%)**			
Ischemic stroke	4/13 (30.8)	4/17 (23.5)	0.698
Hemorrhagic stroke	2/13 (15.4)	0/17 (0)	0.179
Transient ischemic attack	3/13 (23.1)	7/17 (41.2)	0.440
Seizure	0/13 (0)	1/17 (5.9)	1.000
Headache	3/13 (23.1)	4/17 (23.5)	1.000
**Prior diagnosis—no./total no. (%)**			
Hypertension	4/13 (30.8)	4/13 (23.5)	0.698
Diabetes	0/13 (0)	2/17 (11.8)	0.492
Hyperlipidemia	5/13 (38.5)	3/17 (17.6)	0.242
**Lifestyle—no./total no. (%)**			
Smoke	2/13 (15.4)	2/17 (11.8)	1.000
Alcohol	1/13 (7.7)	2/17 (11.8)	1.000
**Drug treatment—no./total no. (%)**			
Antiplatelet drugs	11/13 (84.6)	12/17 (70.6)	0.368
Lipid lowering drugs	7/13 (53.8)	10/17 (58.8)	0.785
Butylphthalide	4/13 (30.8)	4/17 (23.5)	0.698

* *n, number of subjects*.

**Table 2:** Features of hematologic indexes and cerebral blood flow.

**Characteristic**	**Control** (N^*^= 13)	**RIC** (N^*^= 17)	***P*** **-value**
**Baseline**			
**mCBF– ml/100 g per minute**			
Whole brain	47.06 ± 8.44	43.03 ± 8.70	0.213
Cortex	49.11 ± 10.23	45.07 ± 9.40	0.278
Basal ganglia	42.28 ± 10.03	38.28 ± 8.36	0.243
**Hematologic indexes**			
WBC−1,012/L	6.34 ± 2.23	5.63 ± 1.37	0.287
RBC−1,09/L	4.57 ± 0.53	4.64 ± 0.57	0.740
HGB—g/L	133.77 ± 18.57	137.29 ± 16.12	0.583
PLT−109/L	241.77 ± 79.46	234.59 ± 61.93	0.783
ALT—IU/L	28.69 ± 17.05	24.18 ± 12.02	0.402
AST—IU/L	26.38 ± 13.14	23.71 ± 5.64	0.455
CREA—μmol/L	62.62 ± 33.86	52.84 ± 16.15	0.303
UREA—mmol/L	7.62 ± 13.38	52.84 ± 16.14	0.384
CK—IU/L	67.31 ± 35.04	59.21 ± 26.96	0.480
**1-year follow-up**			
**mCBF improvement ratio**			
whole brain	−0.03 ± 0.13	0.16 ± 0.15	0.001
cortex	−0.01 ± 0.13	0.16 ± 0.18	0.007
Basal ganglia	−0.08 ± 0.16	0.15 ± 0.18	0.001
**Hematologic indexes**			
WBC−1,012/L	6.12 ± 1.69	7.76 ± 4.14	0.492
RBC−109/L	4.40 ± 0.26	4.67 ± 0.58	0.444
HGB—g/L	135.25 ± 12.61	138.00 ± 15.17	0.790
PLT−109/L	219.50 ± 97.71	224.00 ± 45.59	0.936
ALT—IU/L	26.75 ± 11.93	19.00 ± 10.20	0.361
AST—IU/L	19.75 ± 2.36	18.25 ± 1.89	0.360
CREA—μmol/L	50.46 ± 11.43	62.30 ± 19.53	0.336
UREA—mmol/L	4.60 ± 1.33	4.64 ± 1.32	0.969
CK—IU/L	68.39 ± 21.83	88.12 ± 58.25	0.549

* *N, number of subjects*.

*mCBF, mean cerebral blood flow; WBC, white blood cell count; RBC, red blood cell count; HGB, hemoglobin; PLT, platelet count; ALT, alanine transaminase; AST, aspartate aminotransferase; CREA, creatinine; UREA, urea nitrogen; CK, creatine kinase*.

**Table 3:** Features of periventricular anastomosis and arterial progression.

**Neuroradiologic outcome**	**Control** (n^*^= 26)	**RIC** (n^*^= 34)	**OR (95%CI)**	***P*** **-value**
**Baseline**				
**Anastomosis—no./total no. (%)**			NA	
Choroidal	6/26 (23.1)	9/34 (26.5)		0.764
Lenticulostriate	3/26 (11.5)	5/34 (14.7)		0.721
Thalamic	3/26 (11.5)	4/34 (11.8)		0.978
MRA score	3.5 (2–5)	3 (3–4)	NA	0.659
**Follow-up**				
Lesion progression—no./total no. (%)	10/26 (38.5)	4/34 (11.8%)	0.21 (0.06–0.79)	0.021
**Anastomosis—no./total no. (%)**			NA	
Choroidal	9/26 (34.6)	9/34 (26.5)		0.495
Lenticulostriate	2/26 (7.7)	5/34 (14.7)		0.402
Thalamic	3/26 (11.5)	4/34 (11.8)		0.978
MRA score	4 (3–5)	3 (3–4)	NA	0.128

* *n, number of hemispheres*.

*MRA, MR angiography*.

### Safety Outcome

No severe adverse events related to RIC occurred in 17 patients of the RIC group during the 1-year treatment. The hematologic indexes reflecting hepatic, renal function, and muscle injury remained normal and no statistical difference was documented (Table 2).

### Efficacy Outcome

#### Neuroradiologic Outcomes

**Figure 2:**
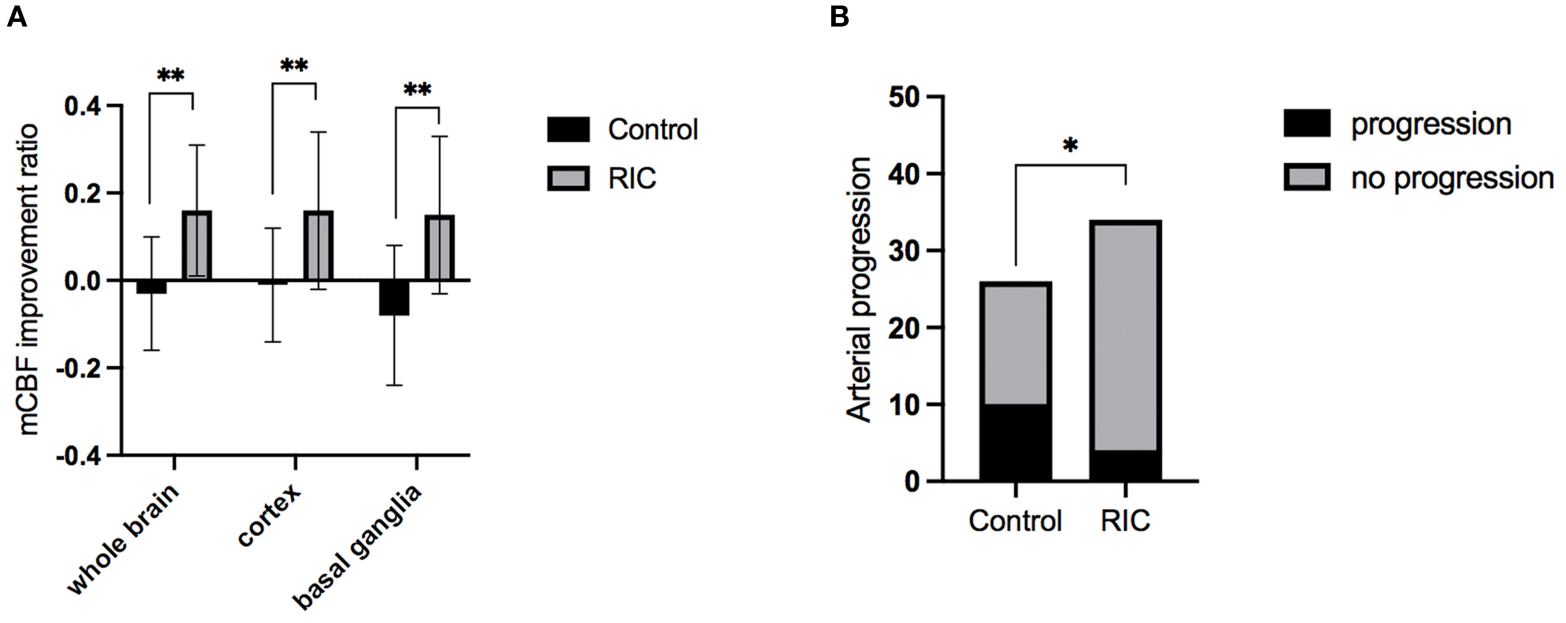
The mCBF improvement ratio and stenotic-occlusive progression of two groups. **(A)** mCBF improvement ratio of whole brain, cortex, and basal ganglia between the two groups at 1-year treatment; **(B)** the number of hemispheres with arterial progression in two groups at 1-year follow-up; mCBF, mean cerebral blood flow; *p < 0.05; **p < 0.01.

The inter-rater agreement was good in evaluating CBF by two radiologists. Intraclass correlation coefficients was 0.942 (95% CI, 0.882–0.972), 0.911 (95% CI, 0.821–0.956), 0.930 (95% CI, 0.858–0.966) for improvement ratio of mCBF_−whole−brain_, mCBF_−cortex_, and mCBF_−basal−ganglia_, respectively. The improvement ratio of mCBF_−whole−brain_ in the RIC group was significantly higher than that in the control group (0.16 ± 0.15 vs. −0.03 ± 0.13, *p* = 0.001). The mCBF of the cortex and the basal ganglia in the RIC group were improved by ratios of 0.16 ± 0.18, 0.15 ± 0.18, respectively, which were significantly higher than those in the control group (*p* < 0.01). The mCBF improvement ratios of the cortex and the basal ganglia in the control group were −0.01 ± 0.13, −0.08 ± 0.16, respectively. In addition, there was no significant difference in the improvement ratio between cortex and basal ganglia in the RIC group (*p* = 0.830). Detailed information is shown in Table 2; Figure 2.

**Figure 3:**
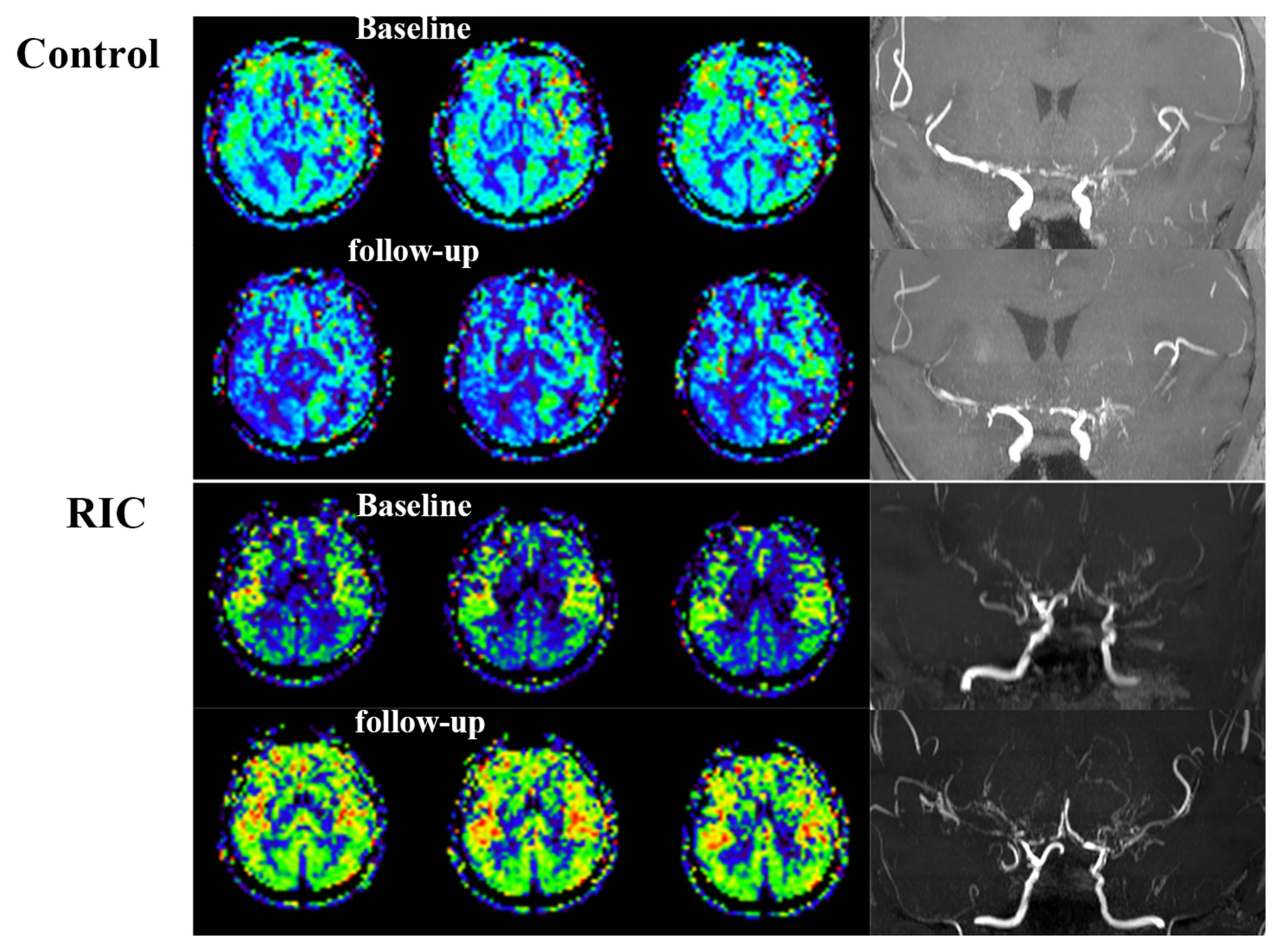
Cerebral blood flow and stenotic-occlusive lesion at baseline and follow-up. ASL images of the patient in the control group showed deterioration of CBF mainly at the right side, which could be reflected by the progression of stenotic-occlusive lesion at right middle cerebral artery (MCA). arterial spin labeling (ASL) images of the remote ischemic conditioning (RIC) group demonstrated improved CBF and more abundant collaterals after RIC treatment in both the sides.

The progression of the stenotic or occlusive lesion in the Willis circle was seen in 10 (38.5%) hemispheres in the control group, while four (11.8%) in the RIC group (OR with RIC, 0.21; 95% CI, 0.06–0.79, *p* = 0.021). In the control group, five hemispheres suffered lesion progression from segment M1 to other segments [four to M2 and one to anterior cerebral artery (ACA)]; three progressed from proximal to distal M1 segment; one progressed from stenotic lesion to occlusive lesion at M1 segment and one progressed from ACA A1 to A2 segment. In the RIC group, three hemispheres suffered progression from segment M1 to other segments (two to M2, one to ACA) and one progressed from proximal to distal M1 segment. The improvement of CBF and stenotic-occlusive lesion of two subjects were shown in Figure 3. No significant difference was documented in MRA score at baseline and 1-year follow-up between the two groups (Table 3). But, we found MRA score of the control group significantly increased after 1-year medical treatment [3.5 (2–5) vs. 4 (3–5), *p* = 0.002], while the MRA score of the RIC group kept stable [3 (3–4) vs. 3 (3–4), *p* = 0.739].

At 1-year follow-up, there were still no differences in three types of periventricular anastomosis between the two groups (*p* > 0.05).

#### Clinical Outcomes

**Figure 4:**
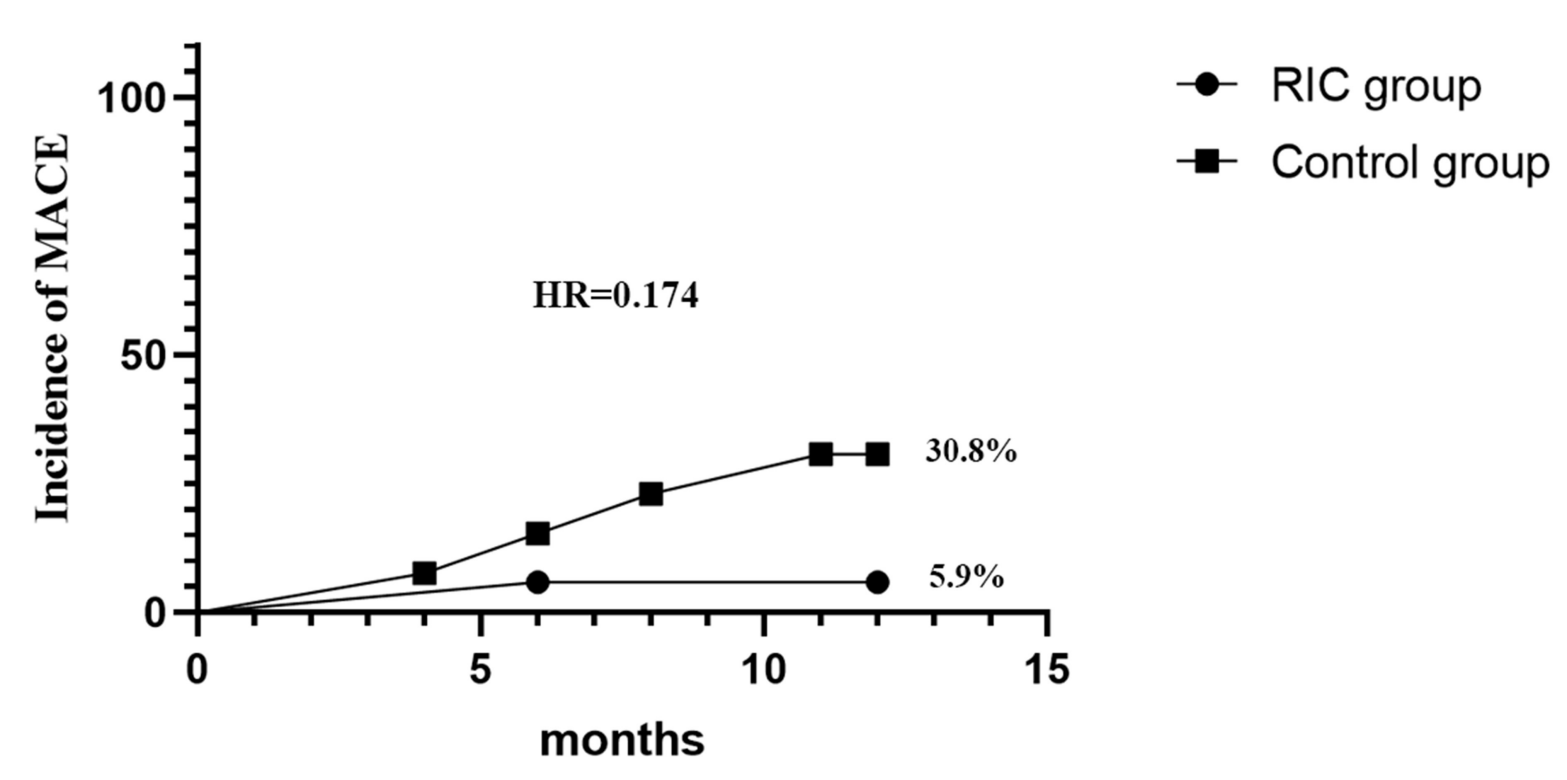
Cumulative incidence of major adverse cerebrovascular events (MACEs) at 12 months.

Major adverse cerebrovascular events occurred in 30.8% of patients in the control group, one suffered TIA, two suffered an ischemic stroke, and one suffered a hemorrhagic stroke. In the RIC group, only one patient (5.9%) suffered TIA and no patients suffered an ischemic or hemorrhagic stroke. However, there was no evidence of a significant difference in cumulative incidence of MACE between the control group and RIC group (hazard ratio with RIC, 0.17; 95% CI, 0.019–1.56, *p* = 0.118) (Figure 4). In addition, there was no distinct difference in any components of MACE containing TIA, ischemic stroke, and hemorrhagic stroke (Supplementary Table I).

## Discussion

Previous studies have shown that daily RIC was well-tolerated and safe in patients with symptomatic ICAS and small vessel disease (12, 13). The present study showed that no patient in the RIC group suffered any RIC-related adverse events and routine blood tests, renal, or hepatic function were normal during 1-year treatment.

Efficacy evaluation indicated that RIC could significantly improve the CBF and alleviate the progression of the stenotic-occlusive lesion in patients with MMD compared with the control group. Besides, the presence of periventricular anastomosis was not statistically different between the two groups. Although non-significantly, there existed a tendency that patients in the RIC group had a lower incidence of MACEs.

Patients with MMD with misery cerebral perfusion have a higher risk of subsequent ischemic stroke (21–23). Recent studies also indicated insufficient perfusion may cause future hemorrhagic events (24, 25). Direct surgery has been generally performed to improve the CBF of MCA territory immediately (26). In addition to improving outcomes of ischemic patients with MMD, a Japan Adult Moyamoya (JAM) trial was conducted to reveal that direct surgery could also reduce rebleeding attacks for hemorrhagic patients (6). The improved perfusion at MCA territory supplied by the external carotid artery system can reduce the hemodynamic burden of choroidal or thalamic collaterals, and then prevents subsequent rupture. Other retrospective studies also indicated a lower risk of cerebral infarction or hemorrhage in patients with MMD after revascularization (27–29). Hence, both cerebrovascular events and improved perfusion are major outcomes that need to be evaluated after revascularization surgery (26).

Thus, it is crucial for patients with MMD to improve and maintain cerebral perfusion of MCA territory. RIC has been shown to improve CBF of patients with chronic circulation insufficiency like ICAS by promoting angiogenesis or arteriogenesis (9–11, 30, 31). A recent single-arm study also observed an improvement of cerebral perfusion in patients with MMD by RIC treatment (14). This study showed that mCBF of the MCA region was improved in the RIC group. The control group conversely tends to have a reduced CBF at 1 year from baseline. Several studies reported that revascularization surgery mainly improved cortical perfusion compared with central regions (32). Our results showed no difference in the CBF improvement ratio between cortex and basal ganglia, which demonstrated that the CBF was increased evenly by RIC treatment. The detailed pathway of improving CBF in patients with MMD by RIC is still not clearly understood. Considering unchanged periventricular anastomosis of basal collaterals after RIC treatment in this study, we assumed that the ischemia tissue may be improved by angiogenesis or arteriogenesis from the leptomeningeal system or external carotid artery system and more stable status of Willis circle. Further studies need to be constructed to validate the assumption by DSA.

The absolute CBF was measured by ASL in this study, which has become a validated technique to monitor CBF changes after revascularization surgery without a contrast agent (26). There is a concern that ASL will underestimate the CBF value in patients with MMD, thus multidelay PCASL-MR that can alleviate the underestimation was utilized (33, 34).

Progressive stenosis or occlusion at Willis circle is one of the characteristics of MMD, which can induce a series of symptoms (35, 36). We evaluated the progression of the stenotic-occlusive lesion and found that patients in the RIC group had a lower risk of arterial progression within 1 year, which may be related to the effects of RIC on improving endothelial function (37), ameliorating inflammation (38), and promotion of arteriogenesis or angiogenesis (9, 10). A recent study showed that RIC could ameliorate vascular remodeling in conduit artery and small resistance artery of hypertensive rats by suppressing deposition of the extracellular matrix and hypertrophy of smooth muscle cells (39). It indicated that RIC may prevent arterial progression through ameliorating vascular remodeling in patients with MMD. But the pattern of vascular remodeling is different between MMD and hypertension, the definite mechanism still needs to be further explored.

The regression of dilated and extended anterior choroidal arteries was seen in patients with MMD after revascularization surgery (40, 41), but it was not found after RIC treatment. The method to evaluate choroidal arteries on non-invasive imaging, relatively short-time follow-up, and small sample size in this study may contribute to the result.

The MACE rate of the RIC group was lower than that in the control group, but it was not statistical. In this study, subjects were mostly composed of the ischemic-onset patients with MMD, and the recurrence rate of ischemic events was reported 3–10% which was relatively low (42, 43). A previous retrospective study with a large sample size also could not get a significant difference in the incidence of recurrent stroke between the surgery group and conservative group until 10-year follow-up (44). Long-term follow-up (3–5 years and even more than 10 years) were commonly used in previous studies observing the efficacy of revascularization surgery (6, 44, 45). Thus, we thought that 1-year follow-up in this study with a small sample size was not sufficient to observe a significant difference in MACE rate between the two groups, and long-term follow-up should be implemented in further research.

Although this study could not determine the effect of RIC on the prevention of cerebrovascular events, the improved CBF of patients with MMD which could be an indicator of better long-term outcome was confirmed. Thus, daily RIC could be an alternative therapy for patients with MMD in the future and will need further study.

This study has some limitations. Small sample size may bring selection bias for this study. There existed a discrepancy in the sample size between the two groups which may be due to the demand for further treatment of patients in the control group without a placebo. The mechanism of RIC for MMD needs further studies to reveal.

Our findings demonstrated that daily RIC treatment is safe and well-tolerated for adult MMD patients. RIC can improve the CBF and halt the progression of stenotic-occlusive lesions. Subjects in the RIC group tended to have a lower risk of MACEs. Thus, RIC seems to be a potential treatment approach for MMD.

## Data Availability Statement

The raw data supporting the conclusions of this article will be made available by the authors, without undue reservation.

## Ethics Statement

The studies involving human participants were reviewed and approved by Ethics Committee of Xuanwu Hospital, Capital Medical University. The patients/participants provided their written informed consent to participate in this study.

## Author Contributions

XJ, SL, QZ, and JX contributed to conceptualization. JX and QZ contributed to writing the original draft. JX, BZ, and WG contributed to the investigation. QY and FW contributed to the methodology. BZ, XX, and WG contributed to data curation. SL, YD, and GR contributed to writing, reviewing, and editing. WZ contributed to the formal analysis. FW contributed to the visualization. JX and SL contributed to project administration. XJ contributed to supervision. SL and XJ contributed to funding acquisition. All authors have read and agreed to the published version of the manuscript.

## Funding

This study was supported by the National Natural Science Foundation of China (No. 81801313) and the Beijing Municipal Administration of Hospitals Incubating Program (No. PX2019028).

## Conflict of Interest

XJ is one of the inventors of the electric autocontrol device that has been patented in China (ZL200820123637.X, China). The remaining authors declare that the research was conducted in the absence of any commercial or financial relationships that could be construed as a potential conflict of interest.

## Publisher's Note

All claims expressed in this article are solely those of the authors and do not necessarily represent those of their affiliated organizations, or those of the publisher, the editors and the reviewers. Any product that may be evaluated in this article, or claim that may be made by its manufacturer, is not guaranteed or endorsed by the publisher.
