# Supplementary material for: Daily Remote Ischemic Conditioning Can Improve Cerebral Perfusion and Slow Arterial Progression of Adult Moyamoya Disease—A Randomized Controlled Study
Source: Front Neurol. 2022 Feb 3;12:811854. doi: 10.3389/fneur.2021.811854 (PMC8850829; doi:10.3389/fneur.2021.811854)
Supplement: Supplementary file 1 [file Data_Sheet_1.docx]

Supplementary Material

# Supplementary Figures and Tables

Figure I. Illustration of regions of interest in MCA territory
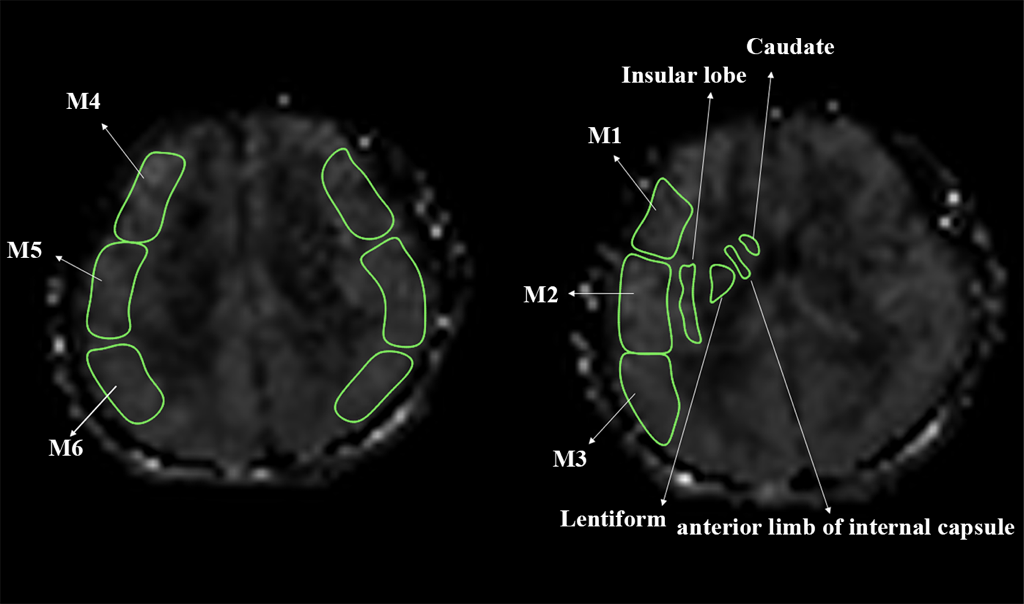


Legends: cerebral blood flow (CBF) of M1, M2, M3, internal capsule, lentiform, insular lobe, caudate was measured in the slice containing basal ganglia. CBF of M4, M5, M6 was measured in the slice including corona radiata.

Table I. Clinical Outcomes and Treatment Effect

| Clinical outcomes | Control  (N*=13) | RIC  (N*=17) | HR (95%CI) | P value |
| --- | --- | --- | --- | --- |
| MACE†—no./total no. (%) | 4/13 (30.8) | 1/17 (5.9) | 0.17 (0.019-1.56) | 0.118 |
| TIA | 1/13 (7.7) | 1/17 (5.9) | 0.74 (0.046-11.75) | 0.827 |
| Ischemic stroke | 2/13 (15.4) | 0/17 (0) | 0.009 (0-1280) | 0.439 |
| Hemorrhagic stroke | 1/13 (7.7) | 0/17 (0) | 0.010 (0-160681) | 0.586 |

*N= number of subjects; †MACE=major adverse cerebral events
